# Time-Sensitive Networking in IEEE 802.11be: On the Way to Low-Latency WiFi 7

**DOI:** 10.3390/s21154954

**Published:** 2021-07-21

**Authors:** Toni Adame, Marc Carrascosa-Zamacois, Boris Bellalta

**Affiliations:** Department of Information and Communication Technologies, Universitat Pompeu Fabra, Carrer de Roc Boronat 138, 08018 Barcelona, Spain; toni.adame@upf.edu (T.A.); marc.carrascosa@upf.edu (M.C.-Z.)

**Keywords:** IEEE 802.11be, low-latency communications, time-sensitive networking (TSN), WiFi 7

## Abstract

A short time after the official launch of WiFi 6, IEEE 802.11 working groups along with the WiFi Alliance are already designing its successor in the wireless local area network (WLAN) ecosystem: WiFi 7. With the IEEE 802.11be amendment as one of its main constituent parts, future WiFi 7 aims to include time-sensitive networking (TSN) capabilities to support low latency and ultra-reliability in license-exempt spectrum bands, enabling many new Internet of Things scenarios. This article first introduces the key features of IEEE 802.11be, which are then used as the basis to discuss how TSN functionalities could be implemented in WiFi 7. Finally, the benefits and requirements of the most representative Internet of Things low-latency use cases for WiFi 7 are reviewed: multimedia, healthcare, industrial, and transport.

## 1. Introduction

The number and type of devices that use Internet to communicate is rapidly increasing, and they are also gaining both complexity and heterogeneity. Within this context, we can find from simple sensor/actuator devices with limited capabilities to high quality video cameras and displays, as well as a myriad of novel wearables ranging from health monitoring devices to Virtual Reality glasses. All of them define what we know as the Internet of Things (IoT) [1].

In recent years, a growing number of heterogeneous productive and entertainment sectors is promoting the development of the IoT towards the support of delay-sensitive applications. This evolution is motivated by the latest technological advances in multimedia, cloud computing, artificial intelligence, automation, robotics, and unmanned vehicles, among many other aspects. They all are fostering the emergence of cutting-edge real-time applications which strongly depend on extremely low latency and, occasionally, very high bandwidth-demanding communications for their successful operation.

Since its emergence in the early 2000s, WiFi’s worldwide success has been mainly substantiated by its high flexibility, mobility of devices, better cost efficiency, and reduced complexity than other solutions. Although WiFi has been constantly evolving through successive amendments to improve peak throughput, capacity, and efficiency, it has not yet been able to produce an effective solution to manage time-sensitive traffic with bounded low latency.

To address the requirements of emerging real-time applications within IEEE 802.11-based networks, initiatives like the Real-Time Application Technical Interest Group (RTA TIG) [2] are promoting physical (PHY) and medium access control (MAC) enhancements, as well as new capabilities under the time-sensitive networking (TSN) framework. Originally intended for Ethernet, TSN sub-standards, which ensure zero packet loss due to buffer congestion, extremely low packet loss due to equipment failure, and guaranteed upper bounds on end-to-end latency [3], are now making their way to wireless networks.

The IEEE P802.11be Task Group (TGbe) [4] was created in May 2019 to address the design of a new PHY and MAC amendment. Considered as the successor of IEEE 802.11ax [5] and the core piece of next WiFi 7, IEEE 802.11be aspires to achieve a peak throughput of 30 Gbps and incorporate disruptive solutions in the WiFi ecosystem such as multi-link operation and multi-access point (multi-AP) coordination [6]. At the same time, IEEE 802.11be also targets reducing worst-case latency and jitter in wireless local area networks (WLANs), for which TSN sub-standards are currently under study for their possible adoption or integration.

Indeed, to be used as part of a potential IEEE 802.11be low-latency operation mode, original TSN mechanisms will need to be redesigned taking into consideration the inherent constraints of the wireless medium (namely, unreliability of links, asymmetric path delay, channel interference, signal distortion, lack of accurate clock synchronization methods, and incompatibility of network interface cards) [7], while ensuring backward compatibility with legacy WiFi devices.

Overall, wireless TSN opens new research directions for the upcoming years, and not only in the WiFi ecosystem [8]. In fact, the 3GPP mobile standards body has also defined ultra-reliable low-latency communications (URLLC) as one of the main application areas for the enhanced capabilities of 5G. Latency reduction techniques and support to deterministic communications have long been in the spotlight of low-power wireless sensor networks, particularly as a result of the specialized MAC-layer profiles introduced in IEEE 802.15.4e.

This paper introduces the future IEEE 802.11be amendment, discussing how its new features can be used to support a seamless adoption of TSN mechanisms. Thus, whereas WiFi networks will never be able to offer bounded delay guarantees due to their own nature and operation in license-exempt bands, the adoption and integration of TSN concepts would keep WiFi as one of the leading wireless access technology in the 6G era, and a key actor to support the increasing needs of the IoT.

The remainder of this article is organized as follows. Section 2 overviews the limitations of current WLANs to handle time-sensitive traffic. Section 3 describes the main features of IEEE 802.11be in terms of PHY and MAC layers. A brief description of TSN and the potential enhancements to support it in WiFi 7 are provided in Section 4. The most representative WiFi 7 use cases that could leverage low-latency communications are reviewed in Section 5. Indeed, the case study of an interactive museum is reviewed in Section 6 to show the benefits of using such a technology. Last, Section 7 presents the obtained conclusions and discusses open challenges.

## 2. Limitations of IEEE 802.11 to Handle Time-Sensitive Traffic

IEEE 802.11 offers great accessibility and ease of use, creating an open environment for any station (STA) willing to associate to the network. However, at the same time, the wireless medium is precisely the main cause that hinders proper delivery of time-sensitive traffic, due to its variable capacity (which depends on the link quality) and typically higher packet error rate (PER; due to the stochastic properties of the channel and the presence of interference) [9].

As for the MAC layer, IEEE 802.11 has traditionally relied on the distributed coordination function (DCF): a contention-based random access scheme based on carrier sense and exponential back-off rules. The main drawback of DCF, however, is its non-predictable behavior and lack of traffic prioritization techniques. In fact, in the presence of multiple STAs, DCF may lead to channel saturation by contending packets, thus being unable to guarantee timely data delivery. The alternative point coordination function (PCF), based on a centralized polling system, has never been widely adopted.

The enhanced distributed channel access (EDCA) was envisioned as part of the IEEE 802.11e amendment to extend DCF and provide quality of service support according to four differentiated access categories (ACs): background, best effort, video, and voice [10]. Prioritization is then implemented by allocating different contention-related parameters to each AC. Nevertheless, the low number of ACs, the lack of mechanisms for the prioritization of different streams belonging to the same AC, and (in some hardware devices) the use of a single buffer to store packets with different priorities are among the main EDCA shortcomings.

To outperform IEEE 802.11e operation for real-time multimedia content delivery, IEEE 802.11aa introduced the intra-AC traffic differentiation functionality, with the definition of two new time-critical voice and video ACs [11]. The capabilities of IEEE 802.11e/aa enhancements to improve the performance of delay-sensitive traffic in WLANs has been widely considered in the literature [12,13,14]. However, in general, none of the IEEE 802.11 mechanisms guarantee the quality of service of heterogeneous real-time streams when a WLAN is overloaded [15]. In such cases, flexible scheduling policies and/or admission control algorithms are highly required to effectively manage different traffic flows.

Neighboring networks represent a key limitation to provide low-latency guarantees in all the aforementioned channel access methods. In dense scenarios, overlapping of basic service set (BSS) coverage areas turns into large delays for STAs waiting to access the channel. IEEE 802.11ax partially addresses this issue by allowing concurrent transmissions under the spatial reuse scope, showing a clear gain for time-sensitive communication [16]. A gain that could be remarkably boosted by means of coordination mechanisms among neighboring APs.

Finally, when it comes to the transport layer, the bufferbloat problem may prevent IEEE 802.11 networks from delivering time-sensitive traffic in presence of TCP flows, due to the high latency produced by excessive buffering of packets. In fact, well-known techniques to mitigate this problem in wired networks (e.g., decreasing buffer sizes and/or applying modern queue management algorithms) have had low success in WiFi [17].

## 3. IEEE 802.11be

This section introduces the main technologies under discussion in TGbe for both PHY and MAC layers and discusses to what extent they would help to satisfy low-latency requirements. In general terms, and following the traditional IEEE 802.11 evolution, IEEE 802.11be will adopt IEEE 802.11ax contributions [5], further refining and extending them, and adding some new features [18,19,20].

### 3.1. PHY Layer

The ongoing release of the 6 GHz band throughout the world will be of great benefit to WiFi dense scenarios, not only due to the additional 1.2 GHz of available spectrum, but also to the resulting interference reduction among networks/BSSs. The incorporation of the 6 GHz band into IEEE 802.11be will also encompass channels as wide as 320 MHz, thus enabling higher transmission rates.

As for the maximum number of spatial streams, it is expected to double its number from 8 in IEEE 802.11ac/ax to 16 in IEEE 802.11be, thus further benefiting from fundamental advantages of predominantly indoor WiFi operation: rich scattering, higher angular spreads, lower correlation, and diversity of channels with good propagation conditions.

The maximum supported modulation size in IEEE 802.11be is likewise expected to be boosted with the adoption of the 4096-QAM modulation, whose practical use, however, will only be feasible in combination with beamforming.

All in all, new IEEE 802.11be PHY features favor low-latency operation, as (1) wider available bandwidth results in faster transmissions and (2) more spatial streams turn into higher rates in the single-user (SU) mode and into more parallel transmissions (with less waiting time in the buffer) in the multi-user (MU) mode.

### 3.2. MAC Layer

Many significant MAC features from IEEE 802.11ax such as MU-MIMO, OFDMA, and spatial reuse will be extended in IEEE 802.11be. The support of more spatial streams will also enable more flexible MU-MIMO arrangements. However, current explicit channel state information acquisition procedure may not cope well with such high number of antennas and, for that reason, TGbe is currently evaluating several alternatives to enhance explicit sounding, even considering the introduction of an implicit procedure.

As for OFDMA, enhanced resource unit (RU) allocation schemes will allow allocating multiple contiguous and non-contiguous RUs to a single STA. Consequently, these novel schemes could significantly increase spectral efficiency and overall network throughput and, even better, satisfy timely data delivery [21]. In fact, whether based on MU-MIMO or OFDMA, MU transmissions are key to reduce the channel access latency, as packets from/to different users can be de-queued simultaneously.

Multi-link operation will likely become the most representative feature of IEEE 802.11be, being able to yield an order-of-magnitude reduction in the worst-case latency experienced by WiFi devices and meet the stringent requirements of real-time applications even under dense traffic conditions [22].

**Figure 1:**
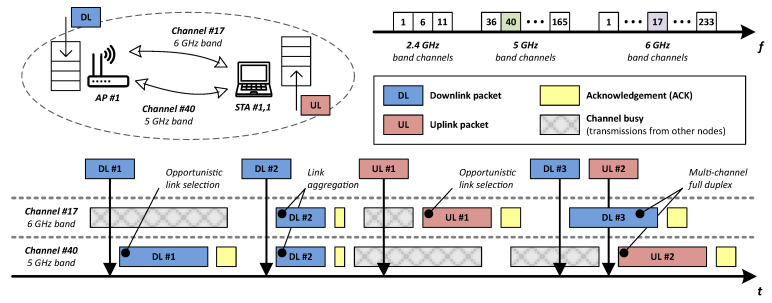
Multi-link operation techniques at ISM frequency bands.

In general, multi-link operation aims to (1) improve throughput by aggregating links [23], (2) enhance reliability by transmitting multiple copies of the same frame in separated links, (3) decrease channel access delay by selecting the first available link in terms of latency [24], and (4) enable isolation of time-sensitive traffic from other network traffic [25]. In short, having two active links operating at different bands/channels between an AP and an STA may increase channel access efficiency by enabling opportunistic link selection, link aggregation, and multi-channel full duplex [26]. Figure 1 shows several of the previously mentioned cases: opportunistic link selection, link aggregation, and multi-channel full duplex.

Two different channel access modes can be considered for multi-link operation: asynchronous and synchronous [27,28].

- Asynchronous mode treats each link individually, allowing both opportunistic link selection (see DL #1 and UL #1 packet transmissions) and simultaneous DL/UL transmissions in a multichannel full duplex fashion (see DL #3 and UL #2 packet transmissions). This mode may create out-of-band emissions, though, resulting in interference between links. Therefore, to operate properly, it requires either large gaps between the selected channels or interference cancellation techniques.
- Synchronous mode is an alternative that avoids interference issues by synchronizing more than one link to transmit at the same time and for periods of equal duration (see DL #2 packet transmission). It uses a primary link that counts the back-off, while the other links are secondary. Once the back-off reaches 0, if the secondary channels have been idle for a PIFS interval they can be used as well [29]. Otherwise, the primary link transmits alone.

TGbe also considers multi-AP coordination, although it is not yet clear if it will finally be included in the final amendment. It allows neighboring APs to share a transmission opportunity and coordinate their transmissions in enterprise-like IEEE 802.11be WLANs, as a way to improve overall performance by means of different techniques:

- Coordinated spatial reuse (CSR) consists in jointly negotiating the transmission power of potential overlapping APs to reduce overall interference. Access delay to the medium could be then reduced, as CSR allows to increase the number of concurrent transmissions.
- Coordinated OFDMA (Co-OFDMA) optimizes the efficiency of the wireless spectrum both in time and frequency, as APs are able to allocate the available RUs to their corresponding STAs in a coordinated way. In consequence, time-sensitive and best-effort traffic could be provided with differentiated RUs to meet timely delivery requirements.
- Coordinated beamforming (CBF) enables simultaneous transmissions from different APs within the same coverage area while ensuring spatial radiation nulls to non-targeted devices [30].
- Distributed MU-MIMO allows APs to perform joint data transmissions (also known as JTX) to multiple STAs by reusing the same time/frequency resources [31]. Spatial diversity can then be exploited to increase frame reception probability.

Thanks to the multi-AP coordination, multiple overlapping BSSs (OBSSs) can turn channel contention in their favor, resulting in a better use of shared resources. Beyond the latency reduction obtained by using the spectrum more efficiently, new solutions to protect time-critical traffic across the cooperating BSSs may be enabled. For instance, APs dealing with best-effort traffic may agree on reducing transmission power to provide spatial reuse opportunities, so that STAs from other BSSs can successfully transmit their short-duration, time-sensitive packets at the same time.

Advanced transmission schemes such as hybrid automatic repeat request (HARQ) offer notable performance gains in varying channels compared to the traditional stop & wait approach, but it is not yet clear if such gains will also be achieved in WLANs due to the severity of collisions. Be that as it may, HARQ still retains the goal of reducing average latency, because of its performance improvements in PER [32].

In short, the new IEEE 802.11be MAC functionalities will help to use more efficiently the spectrum resources and allocate them in a more flexible way to optimize throughput, latency, or reliability, depending on the scenario requirements. Furthermore, these functionalities can be better exploited for low-latency purposes if some core TSN features (e.g., admission control and scheduled operation) are integrated on top of them, as we will see in Section 4.

Last but not least, the lack of legacy devices operating in the upcoming 6 GHz band also offers the possibility of rethinking channel access for future WiFi 7 adopters. In this sense, traditional channel access schemes based on contention might be partially replaced by others able to offer higher levels of determinism, thus facilitating the management of real-time deterministic traffic and the inclusion of TSN mechanisms.

### 3.3. Standardization Status

The standardization process of IEEE 802.11be, initiated by TGbe in May 2019, consists of two stages: Release 1 and 2, and it is expected to be completed in May 2024 with the publication of the final amendment. Release 1 is aimed to prioritize the development of a small distinctive set of IEEE 802.11be candidate features, such as the 320 MHz channels, the 4096-QAM modulation, and the multi-link operation, becoming available by 2022. Release 2 shall contain the rest of the features (maybe including also a low-latency operation mode) as well as the potential extensions and/or modifications of the already introduced ones in Release 1.

## 4. Supporting TSN in WiFi 7

TSN consists of a set of sub-standards defined by the IEEE 802.1 TSN Task Group [33] to support deterministic messaging on standard Ethernet. Therefore, a single network with TSN capabilities can be shared by time-sensitive and best-effort applications. Essentially, TSN technology relies on a central management solution that uses time scheduling to ensure reliable packet delivery with bounded latency and low packet delay variation (*jitter*) in deterministic real-time applications.

In principle, TSN capabilities can be mapped seamlessly from Ethernet to WiFi, without architectural changes nor protocol translation gateways. However, the proper operation of TSN in WiFi and, in consequence, the meeting of time-sensitive requirements, is mainly hindered by (1) the variability of wireless channel conditions (even without device mobility), (2) the interference proneness in unlicensed spectrum bands, and (3) the inherent randomness of medium access in WiFi.

A careful design of WiFi 7 technologies taking into account the TSN principles and the wireless specificity could certainly contribute to reduce WiFi latency issues, yet at the present time that potential integration is neither straightforward nor exempted from uncertainties and incompatibilities. Therefore, the approach followed by the TGbe to prove the feasibility of TSN over wireless could encompass already available IEEE 802.11 mechanisms, native versions or adaptations from TSN sub-standards, and proposals of new solutions in several areas, as compiled in Table 1.

This section elaborates on some key components of TSN, analyzes the most suitable IEEE 802.11be enhancements to support them, points out the main challenges involved in the integration process, and sheds some light on possible solutions and open research directions.

### 4.1. Network Configuration

The IEEE 802.1Qcc TSN sub-standard defines a set of management interfaces and protocols to enable TSN network administration. It allows standard, consistent setting of policies to manage the available network resources (e.g., communication paths, bandwidth, scheduling patterns, and so forth) with the aim of meeting time-sensitive needs of end applications.

**Figure 2:**
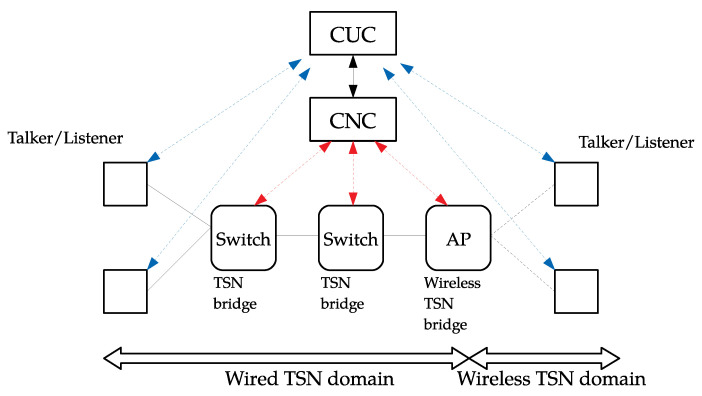
Centralized network management model for wired-wireless TSN networks.

As it can be observed in Figure 2, wireless TSN networks are expected to seamlessly extend the wired TSN domain, with which will share two main management entities in an SDN-inspired centralized scheme:

- The centralized user configuration (CUC), which receives requirements from users (blue lines) of TSN network services (talkers and/or listeners) and communicates them to the central network controller (CNC).
- The central network controller, which receives consolidated requirements from the CUC. Then, it is responsible for determining routes, scheduling TSN flows through the network, and configuring TSN-enabled bridges accordingly (red lines).

Under this scheme, APs can be seen as wireless TSN bridges configured by the CNC, whereas STAs act as talkers or listeners with permanent communication with the CUC. To cope with the variability introduced by fading and mobility, TGbe is currently considering multiple wired–wireless TSN integration and configuration models, resulting from the balance between network management complexity and efficiency [34].

### 4.2. Time Synchronization

The IEEE 802.1AS TSN sub-standard includes a version of the precision time protocol (PTP), which enables the distribution of a single reference clock across network devices in a master/slave basis. The availability of a common clock is likewise a key requirement for future wireless TSN-enabled technologies like WiFi 7, as it would permit to successfully schedule MU transmissions in both uplink and downlink, as well as to establish coordination mechanisms among APs [35,36].

Indeed, IEEE 802.1AS can already be operated over IEEE 802.11 by means of the timing measurement (TM) procedure defined in IEEE 802.11v, which takes wireless link asymmetric delay into consideration. Time is propagated in private action frames between a master (i.e., the AP) and a slave (i.e., the STA), with the latter being able to compute the clock offset and adjust its own time accordingly.

Furthermore, the last revision of the IEEE 802.1AS standard (IEEE 802.1AS-2020 [37]) contains the fine timing measurement (FTM) procedure, a novel synchronization method already included in IEEE 802.11mc. FTM can be considered an extension of the TM scheme, but with a finest timestamp resolution (0.1 ns vs. 10 ns) and some other minor changes that do not hamper backwards compatibility [38].

### 4.3. Traffic Filtering and Policing

IEEE 802.1Qci per-stream filtering and policing (PSFP), also known as ingress policing/gating standard, allows filtering and policing individual traffic streams based on rule matching. This TSN sub-standard protects time-sensitive flows from excessive bandwidth usage, burst sizes, incorrectly configured endpoints, and malicious attacks [39].

In short, thanks to their identifier, streams are first filtered according to per-stream policies. Then, a gating mechanism regulates the stream. Finally, stream metering ensures bandwidth limitations before a frame is queued for forwarding. To the best of our knowledge, no adaptations of IEEE 802.1Qci are included in the TGbe roadmap. However, the necessity of a potential extension of this TSN sub-standard to wireless networks has already been pointed out in previous related work [25].

A traditional approach in IEEE 802.11 to protect time-sensitive traffic is to avoid channel overloading (i.e., to limit the traffic load, the number of traffic flows, and/or the number of STAs allowed to transmit data) in a given band and time period:

- Whereas IEEE 802.11e admission control mechanisms limit the number of traffic flows per service class in a BSS, IEEE 802.11aa extends this capability to an entire OBSS. Both could be used by WiFi 7 alone or in combination with IEEE 802.Qci-inherited policies to control how traffic arrives to transmission buffers.
- Multi-link operation and the incorporation of the 6 GHz band in IEEE 802.11be foresee the emergence of traffic-aware multi-band admission control and policing systems. For instance, under the asynchronous mode of the multi-link operation, and depending on the network conditions and existing load, the 6 GHz band could be fully and exclusively dedicated to time-sensitive traffic. Several multi-link load balancing policies are introduced and evaluated in [28], showing that distributing the traffic over the different interfaces may not be the best approach as it makes the traffic more vulnerable to the activity of the neighboring BSSs.

Future admission control mechanisms may also be required to support scheduled operation. Thus, in the aforementioned example, the 6 GHz band would become even more exclusive, by only accepting time-sensitive traffic coming from devices able to operate in contention-free mode.

### 4.4. Traffic Classification

IEEE 802.1Q standard specifies up to eight different traffic classes by means of the priority code point (PCP) in the VLAN tag, which helps to identify and differentiate time-sensitive traffic. In concordance, bridges supporting IEEE 802.1Q allocate packets to outbound queues according to their traffic class. Then, if no other TSN traffic scheduling (e.g., IEEE 802.1Qbv) or shaping method (e.g., IEEE 802.1Qav) is used, a strict priority policy first forwards packets from queues with higher traffic class values.

IEEE 802.11-2016 specification supports VLAN tag traffic stream differentiation as defined in IEEE 802.1Q, by means of the traffic specification (TSPEC) and traffic classification (TCLAS) elements (the IEEE 802.11 header includes the traffic ID (TID) field to classify the type of traffic. TID is used to select a user priority (UP) for prioritized QoS or a TSPEC for parameterized QoS. Whereas TID values between 0 and 7 are considered UPs, those between 8 and 16 refer to TSPECs). In consequence, appropriate traffic classification mapping enables seamless interworking among wired and wireless networks [40].

As for the EDCA access categories (ACs), they map directly from the aforementioned Ethernet-level traffic classes. However, having only four ACs is insufficient for fine control of real-time applications, as they cannot provide hard bounds on latency/jitter, especially under congestion. For that reason, TGbe is also considering possible enhancements to EDCA, such as the incorporation of a new AC with the highest priority for time-sensitive traffic [41], as well as some modifications to how obtained transmission opportunities (TXOPs) are used (for instance, to allow using any TXOP, regardless the AC that has obtained it, to send time-sensitive traffic when available [20]).

### 4.5. Scheduled Operation

IEEE 802.1Qbv TSN sub-standard creates a time division multiple access (TDMA) scheme that splits communication time on an Ethernet network into repetitive cycles of fixed length. A time-aware scheduler defines the time period in which gates corresponding to each outbound queue (each one linked to a different traffic class) are opened or closed. In consequence, time-sensitive frames can be transmitted with the certainty that they will not be interfered by other best-effort traffic [42].

**Figure 3:**
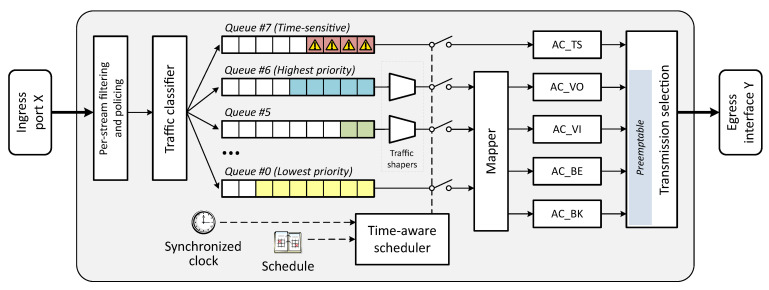
Diagram of the integration of several time-sensitive enhancements into a potential wireless TSN AP.

The potential adaptation of the IEEE 802.1Qbv time-aware scheduler on top of one of the IEEE 802.11 MAC modes would allow devices to control how traffic arrives to the different EDCA ACs according to new rules yet to be defined. By scheduling the traffic that arrives to the MAC layer, devices will be able to reduce inter-AC contention, as well as better control how and when they content for the channel. On this basis, Figure 3 exemplifies a hypothetical integration and joint operation of a wireless TSN AP using a time-aware scheduler in combination with EDCA.

Isolated transmission of time-sensitive traffic on a periodic basis has already been explored by IEEE 802.11. In this sense, two methods of scheduled access facilitating collision-free operation are available in IEEE 802.11ax:

- The trigger-based access allows the AP to schedule uplink MU transmissions by means of MU-MIMO, OFDMA, or both joint techniques [43]. In any case, unfairness is likely to appear in heterogeneous networks with coexisting IEEE 802.11ax and legacy devices, as the former can access the medium only after the AP gets the TXOP. Even so, this problem could be alleviated by applying EDCA appropriately and setting different transmission priorities to nodes [44]. Last, as a potential direction to reduce signaling overhead and support lower latency bounds in trigger-based access, TGbe has identified the use of persistent UL allocation schemes [45].
- By using the target wake time (TWT) mechanism, STAs adopt a wake time schedule that makes them wake up on a periodic basis to transmit/receive data [46]. However, when a new TWT service period starts, there is no guarantee another device that have gained a TXOP just before overlap in time with the TWT service period. To mitigate this situation, and further protect scheduled traffic, IEEE 802.11be will include an extension of TWT called Restricted TWT, which simply forces all other IEEE 802.11be-compatible devices to finish their transmissions before the TWT service period begins.

Whereas the two aforementioned methods just determine the very moment in which the channel is accessed, the new MAC features fostered by TGbe could empower scheduled operation, especially if, as discussed before, only devices supporting those mechanisms are admitted in the 6 GHz band. In consequence, multi-link operation and OFDMA could play an important role by allocating devices’ and network resources together with the computed schedule in function of the existing time-sensitive traffic load.

**Figure 4:**
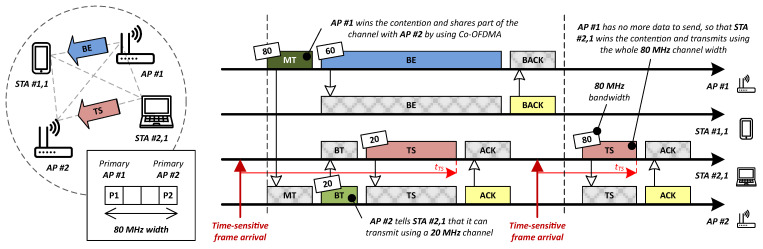
Multi-AP resource coordination based on Co-OFDMA; MT: Multi-AP trigger, BT: Basic trigger, BE: Best-effort frame, TS: Time-sensitive frame, (B)ACK: (Block) acknowledgment, and $t_{\mathrm{TS}}$: Time-sensitive frame latency. Note that this example is a proposal from the authors to illustrate how multi-AP coordination may work, and so there is no direct correspondence with any working document from the IEEE 802.11be Task Group.

In brief, such a scheduled operation is key in terms of delay. However, the main obstacle that hinders its precise operation in the wireless domain continues to be the contention in the context of several OBSSs, which can only be effectively handled in combination with a proper multi-AP resource coordination strategy. By way of an example, Figure 4 shows how the use of Co-OFDMA may help make a more efficient use of the available channel resources while meeting time-sensitive requirements.

In Figure 4, two different frames originated in the same BSS (a best-effort one from AP #1 and a time-sensitive one from STA #2,1) are simultaneously transmitted by splitting the total 80 MHz spectrum bandwidth into two non-overlapping channels of 60 MHz and 20 MHz, respectively. To make this coordination possible, once AP #1 wins the contention, it signals the RU allocation information by means of a multi-AP trigger. In turn, AP #2 notifies STA #2,1 of its corresponding RUs with a basic trigger.

### 4.6. Traffic Shaping

Besides the time-aware scheduler, TSN offers additional prioritization mechanisms in form of traffic shapers. They are used to manage the bandwidth of a stream to comply with a predefined traffic profile by pacing the output of packets (e.g., in a media stream). Three different TSN sub-standards compile the standardization activities regarding traffic shapers:

- IEEE 802.1Qav credit-based shaper ensures provision of maximum required bandwidth for media streams without a noticeable interruption of best-effort traffic. Time-sensitive packets are evenly distributed over time by means of a leaky bucket credit-based fair queuing.
- IEEE 802.1Qch cyclic queuing and forwarding collects packets according to their traffic class in one cycle and forwards them to the next hop in the subsequent cycle.
- IEEE 802.1Qcr asynchronous traffic shaping is the only one that operates asynchronously, aiming to provide deterministic and relatively low transmission delay for general time-sensitive flows according to an urgency-based scheduler [47].

As for the potential integration of TSN-based traffic shapers into the IEEE 802.11 ecosystem, note that the credit-based traffic shaping mechanism (i.e., IEEE 802.1Qav) could be straightforwardly adapted to the IEEE 802.11ax scheduling mode provided that the latter was able to ensure the required periodic TXOPs to achieve a given bandwidth [25].

Multi-link operation in IEEE 802.11be can also benefit from traffic shapers to efficiently allocate several packets of a stream into the available network interfaces at a given time. For instance, the synchronous mode allows for a group of aggregated packets to be split between the different active links, thus reducing the total transmission time of aggregated packets. In this regard, careful shaping considering each link’s availability could help reduce the overall delay of the network.

### 4.7. Transmission Selection

IEEE 802.1Qbu TSN sub-standard implements frame preemption to interrupt the ongoing operation of a low-priority (preemptable) queue if a time-sensitive (preempting) queue is selected for transmission. In addition, low-priority frames are split into smaller fragments to further reduce overall latency.

**Figure 5:**
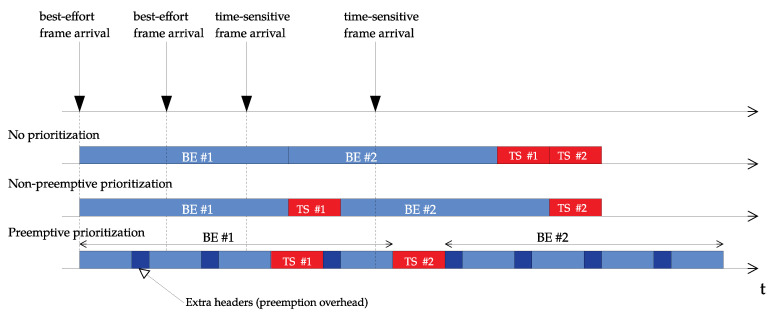
Frame preemption mechanism [48], being BE: Best-effort frame, and TS: Time-sensitive frame.

If a wireless device is transmitting multiple traffic flows, placing the time-sensitive traffic in the highest priority queue may not be enough to mitigate the residual delay caused by large ongoing low-priority transmissions, which may include many aggregated packets and last up to the maximum physical protocol data unit (PPDU) duration (i.e., ∼5 ms). As shown in Figure 5, a possible solution to that issue could be based on the adaptation of the IEEE 802.1Qbu frame preemption mechanism, which would also foster the use of packet aggregation for best-effort traffic even in presence of time-sensitive traffic, thus improving overall throughput.

Just as IEEE 802.3br does it with respect to Ethernet, integrating frame preemption into WiFi would require several changes in the physical and link layers, such as the format of preemptable frames and the methods to fragment frames while preserving integrity of preemptable traffic. In any case, it seems reasonable to only support this new feature when aggregate MAC protocol data units (A-MPDUs) are transmitted, and so extend the service field used to identify the different MPDUs.

Despite the fact that frame preemption may well be applied on outgoing transmissions from the same node, its extension to incorporate transmissions from other APs/STAs would require a complex channel access mechanism. In that situation, and in presence of a time-sensitive traffic flow, it would be advisable to simply avoid the use of packet aggregation in the OBSS, even if that implied a severe throughput loss.

### 4.8. Ultra-Reliability

Ultra-reliability in TSN is responsibility of the IEEE 802.1CB frame replication and elimination for reliability (FRER) sub-standard, which sends duplicate copies of each frame over disjoint wired paths to provide proactive seamless redundancy. To minimize network congestion, FRER can also be applied only on determined traffic classes (e.g., critical or time-sensitive traffic) and paths.

The potential incorporation of IEEE 802.1CB into the IEEE 802.11 ecosystem relies on the IEEE 802.11ak amendment, which is able to create link-disjoint or node-disjoint paths. However, to actually improve wireless path reliability TGbe is considering other complementary enhancements [49]:

- Multi-link operation (i.e., frequency diversity): use of separated bands to transmit the same frame between multi-link devices. In fact, a first testbed has proven the latency reduction associated to the transmission of the same time-sensitive traffic flow over two different channels in presence of interfering traffic [50].
- Multi-AP operation: use of joint transmission (JTX) from different APs to improve reception probability in the DL. Particularly, JTX provides a better performance gain compared to other multi-AP transmission schemes at the expense of adding complexity for synchronization [51].

## 5. Use Cases

The ability of WiFi 7 to support low-latency operation would open the door to multiple IoT use cases. This section groups them into a set of productive sectors, details their performance requirements in Table 2, and discusses the suitability of using WiFi 7 with respect to other alternatives.

### 5.1. Multimedia

WiFi is nowadays the predominant Internet access technology for mobile devices in home and office environments running multimedia applications. The short-term evolution of Multimedia IoT foresees the consolidation of more advanced time-sensitive services such as real-time high-quality 4K/8K audio and video streaming, virtual reality, augmented reality, cloud gaming, and interactive applications which will not be only targeted for entertainment, but also for educational and instructive purposes [52].

**Figure 6:**
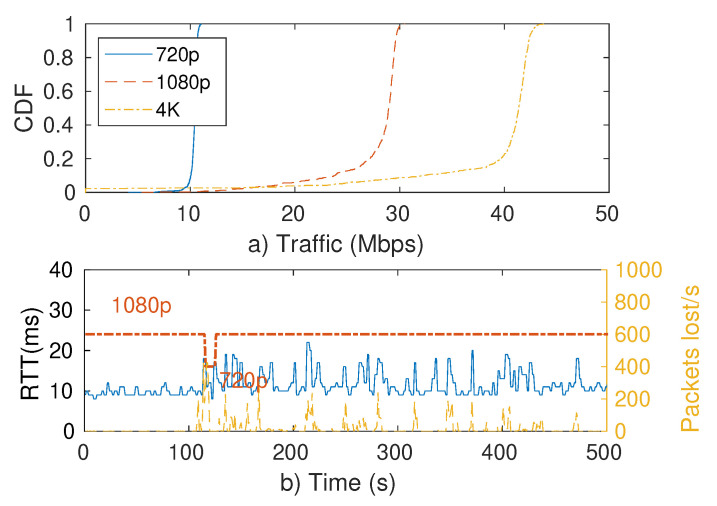
(**a**) Google Stadia’s Tom Raider traffic load according to video resolution and (**b**) Measured RTT when the network has a bandwidth limit of 30 Mbps (dashed line corresponds to video resolution).

Indeed, 60.6% of all downlink Internet traffic already corresponds to video streaming, while online gaming takes another 8% [53]. New cloud gaming applications like Google Stadia and Nvidia Geforce Now combine these two categories by streaming videogames directly from a remote server to the user. These applications cannot buffer due to their interactive nature, and so they require high throughput (Figure 6) as well as low delay, having issues when round-trip time (RTT) goes above 10 ms [54].

Moreover, studies show that the brain can identify images seen for as little as 13 ms [55], which is less than the time duration of a single frame of video at 60 fps (i.e., 16.7 ms). Current online game companies (and potentially in the future other online video content distributors) use precisely this framerate in their streamings, which imposes stringent requirements on delay and reliability.

All in all, the already generalized adoption of WiFi technology indoors, its backward compatibility, and its distinctive features with respect to wired alternatives (that is, essentially, flexibility, simplicity, and mobility) suggest that the emergence of a low-latency operation mode for WiFi 7 would position it as a preferential option for upcoming multimedia use cases together with 5G enhanced Mobile Broadband.

### 5.2. Healthcare

Since the Smart Health concept was coined as a context-aware health paradigm within Smart Cities [56], the adoption of information and communication technologies (ICT) within the healthcare sector has unstoppable grown over the years [57]. Current well-established use cases emerged from this IoT-based concept, such as remote health monitoring, patient identification and tracking, drug management, or hospital asset management [58].

Latest IT advances such as ultra high video resolution, Big Data, and artificial intelligence will take Smart Health to a next level, enabling a plethora of innovative applications in remote diagnosis (telediagnosis), treatment (telesurgery), and recovery (telemonitoring, telerehabilitation, exoskeletons, and prosthetic hands) for a wide set of diseases. WiFi 7 and 5G will here again play an important role as enablers of novel medical wearables and devices intended for use in smart health care and home environments.

As for the specific use cases, some of them share common characteristics with the multimedia sector (e.g., telediagnosis, telemonitoring, and telerehabilitation). Due to their criticality, some others additionally impose extremely stringent network requirements in terms of end-to-end reliability, latency, and security (e.g., telesurgery). Last, a third group involving remote motion control with relatively low traffic load requires a fully deterministic approach (e.g., exoskeletons and prosthetic hands).

### 5.3. Industrial

During the next years, wireless networks will have increasing weight in the industry, leading a trend towards more flexible production sites. The Industry 4.0 concept, based on the cyber-physical transformation of processes, systems, and methods of manufacturing in the industrial sector, will enable autonomous and decentralized operation while ensuring proper coordination with commercial and logistics systems.

One of the multiple existing Industry 4.0 applications is known as connected factory, involving monitoring, management, and direct control of machines, robots, and other industrial assets. The critical nature of some specific manufacturing processes, with typical latency requirements from 1 to 200 ms, makes crucial to guarantee reliability with determinism; that is, that each message must reach its destination within its scheduled period [59].

Future industrial communications will probably rely on the coexistence among wired (e.g., Fieldbus-based and Industrial Ethernet), wireless (from RFID to LoRa, to cite two examples), and 5G/6G-based cellular technologies. WiFi 7 is also expected to get a foothold in this sector, not only because of its inherited features (namely, flexibility, ease of installation, scalability, and interoperability), but also thanks to its new enhancements, particularly in terms of improved resource management and support to deterministic communications.

### 5.4. Transport

Transport is experiencing such profound changes that future mobility will certainly be substantiated by automation, sustainability, road/air/sea safety, and energy efficiency. Real-time traffic information is starting to be served on a regular basis to drivers, using for instance city-wide WiFi deployments. Yet the upcoming revolution is being led by autonomous vehicles and automated guided vehicles, which will be able to transport people and goods thanks to their WiFi/5G connections without any human intervention.

Next-generation vehicle communication and processing systems, such as vehicle-to-everything communication or advanced driver-assistance systems, will assist future transport systems on the basis of TSN and artificial intelligence. Therefore, ensuring very high reliability and low latency in future transport applications will become crucial regardless the employed technology, due to the high relative speeds among end devices, and the continuous dynamism and low predictability of the outdoor environment.

## 6. Case Study: Interactive Museum

Worldwide museums have long relied on IoT-related technologies to display information, give context, and involve visitors in their exhibitions. Well-known examples are informational videos, audio guides, interactive games, hands-on experiments, and smartphone apps. In this regard, the latest advancements on augmented reality (AR) allow its adoption by interactive museums, thus giving curators a chance to layer more information on top of existing exhibits [60].

To cope with the volume, distribution, and dynamic behavior of visitors across the different halls (usually moving far and wide and even creating densely populated clusters of people), the museum’s wireless network does not only require the deployment of a high number of APs, but also a coordinated operation under a multi-AP scheme.

In the following lines, the case study of an interactive museum is used to illustrate the benefits of integrating a TSN feature such as frame preemption into IEEE 802.11be. MATLAB is used to simulate the considered scenario on the basis of parameters from Table 3.

**Figure 7:**
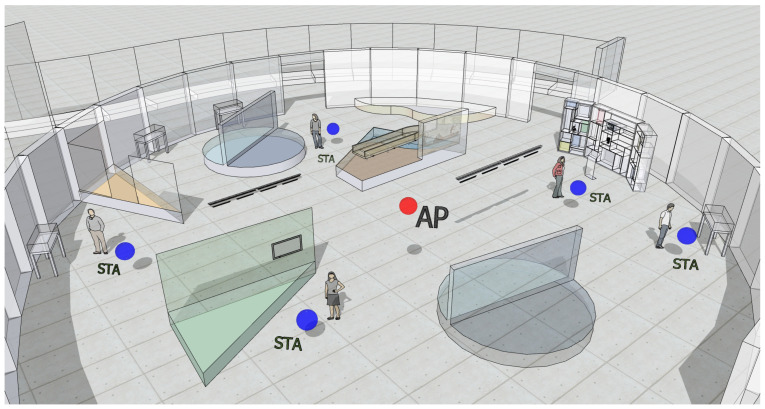
Interactive museum 3D view.

Let us consider a circular hall of radius R=15 m containing an interactive exhibit with a single AP placed in the center, as shown in Figure 7. (Image based on the Google SketchUp model of the Museum Mysteries exhibit of the Waterloo Region Museum located in Kitchener, Canada (https://3dwarehouse.sketchup.com/model/ufac3aabd-f3c2-4caa-b775-785217b2e9e8/Museum-Mysteries-Exhibit, accessed on 20 July 2021)). Users are placed uniformly at random on the museum’s hall. The AP provides visitors in that hall with a set of customized interactive services by means of DL unicast transmissions that can be categorized according to their priority level:

- Best effort (BE) traffic is tagged as low-priority and consists of video streaming of additional contents, an interactive audio guide, and real-time information feeding the museum mobile app. BE traffic amounts to $B_{\mathrm{BE}}=2$ Mbps per user.
- Time-sensitive (TS) traffic is tagged as high-priority and transports a stream of information corresponding to an immersive AR installation. TS traffic amounts to $B_{\mathrm{TS}}=\;5$ Mbps per user and has a maximum tolerable latency of $t_{\mathrm{TS}}=5$ ms.

The AP implements two access categories (BE and TS), and supports three different prioritization policies:

- No prioritization: Packets are sent in strict order of arrival regardless their type (BE or TS). Are all the italisc necessary? If not, please reivse.
- Non-preemptive prioritization: As long as the TS queue contains packets, they are sent prior to BE ones. In case there is an ongoing transmission of a BE packet, and a new TS packet arrives to the TS queue, the transmission of the latter is delayed until the end of the former one.
- Preemptive prioritization: As in the previous policy, TS packets are sent prior to BE ones. However, the transmission of a BE packet is interrupted if a new TS packet arrives. Transmission from the BE packet is resumed only when the transmission of the TS packet finishes and the TS queue is empty. Preemption, however, entails an extra delay caused by overheads of the $N_{f}$ fragments in which the BE packet is divided ($N_{f}\cdot T_{ov}$).

Visitors may request low-priority or high-priority services, or even both at the same time. Four different network configurations were considered, being $N_{\mathrm{BE}}$ = $\left\{15,20,25,30\right\}$ users requesting BE services and $N_{\mathrm{TS}}=5$ users requesting TS services. For each configuration, k=1000 simulations changing user locations were executed. Then, according to the TMB path loss model for 5 GHz indoor scenarios [61] and the MCS table from IEEE 802.11ax (it is expected that IEEE 802.11be inherits the MCS table from IEEE 802.11ax and enriches it with more modes derived from its enhancements in the PHY layer: up to 16 spatial streams, higher modulations (4096-QAM), and wider channels (320 MHz)), data rate of each user was automatically computed.

**Figure 8:**
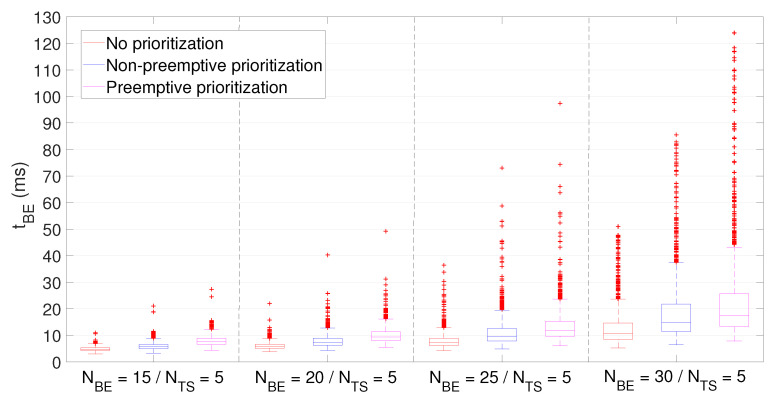
Interactive museum use case simulation. Average latency of BE packets.

**Figure 9:**
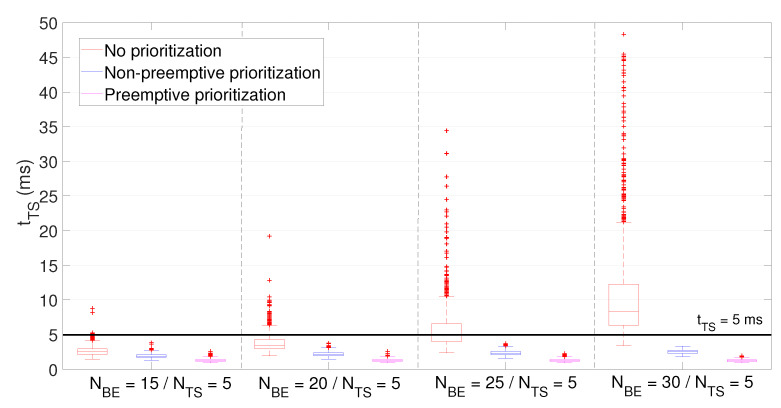
Interactive museum use case simulation. Average latency of TS packets.

Average latency of BE and TS packets (shown in Figure 8 and Figure 9, respectively) was computed for the three aforementioned priority policies and the four different network configurations (based on the number of $N_{\mathrm{BE}}$ and $N_{\mathrm{TS}}$ users). As expected, those policies giving priority to TS packets resulted in a reduced latency for the time-sensitive traffic.

If we observe the latency of BE packets, it grows in all three policies when increasing $N_{\mathrm{BE}}$, attaining the preemptive prioritization policy the highest values but with a median contained below 20 ms. Moreover, although outliers achieve up to 125 ms in some concrete cases, they are always below 150 ms. As for latency of TS packets, its value grows in line with $N_{\mathrm{BE}}$ when using the no prioritization policy, unable to meet the stated requirement (i.e., $t_{\mathrm{TS}}$ < 5 ms) on average for more than $N_{\mathrm{BE}}=20$ users.

On the contrary, non-preemptive and, especially, preemptive prioritization policies are able to keep latency values regardless $N_{\mathrm{BE}}$ below 3.83 ms and 2.58 ms, respectively. In addition, these values attain very low dispersion, thus ensuring predictable bounded latency and, therefore, supporting time-sensitive communications.

## 7. Conclusions

A new world of technological possibilities could make its way in a very varied range of IoT sectors thanks to the integration of TSN and the well-established IEEE 802.11 technology. The most solid and promising exponent of this trend is IEEE 802.11be, actual precursor of future WiFi 7, which should be accompanied with a well-defined and backward compatible time-sensitive operation mode to support low-latency communications.

Although WiFi will never be able to guarantee fully deterministic communications because of its operation in license-exempt bands, there is still room to reduce the impact of all manageable causes, both internal and external, that may increase latency. On the one hand, contention with external networks may be minimized by considering dynamic spectrum access such as non-contiguous channel bonding and multi-link operation, as well as cooperative AP strategies. On the other hand, prioritization and scheduling mechanisms inside the same WLAN may provide an effective solution to reduce the latency of time-sensitive traffic in the presence of large packets from best-effort flows.

## Figures and Tables

**Table 1:** Potential enhancements to support TSN requirements in WiFi 7.

Component	TSN Sub-Standard	Status	Targeted Features
**Network configuration**			
IEEE 802.1Qcc	✓	S	M
**Time synchronization**			
IEEE 802.1AS over IEEE 802.11	✓	A	J, L
IEEE 802.11mc FTM		A	J, L
**Traffic filtering and policing**			
IEEE 802.1Qci	✓	-	L, R
IEEE 802.11e/aa admission control		A	L, R
Multi-band admission control		S	L, R
**Traffic classification**			
IEEE 802.1Q-based VLAN tag traffic stream differentiation (TSPEC, TCLAS)		A	C
EDCA operation enhancements		S	L
**Scheduled operation**			
IEEE 802.1Qbv-based time-aware scheduler	✓	S	J, L, R
IEEE 802.11ax trigger-based access		A	J, L, R
IEEE 802.11ax TWT mechanism		A	J, L, R
Multi-AP coordination		R2/S	J, L, M, R
RTWT		R1/S	J, L, R
**Traffic shaping**			
IEEE 802.1Qav credit-based shaper	✓	S	J, L
IEEE 802.1Qch cyclic queuing and forwarding	✓	-	J, L
IEEE 802.1Qcr asynchronous traffic shaping	✓	-	L
**Transmission selection**			
IEEE 802.1Qbu-based frame preemption	✓	S	J, L
**Ultra reliability**			
IEEE 802.1CB over IEEE 802.11ak	✓	A	R
Multi-link operation		R1	L, R
HARQ		S	L, R

Status: (A) already available in IEEE 802.11, (R1) in the roadmap of IEEE 802.11be Release 1, (S) under study by TGbe, (-) not considered by TGbe. Targeted features: (C) traffic classification, (J) jitter, (L) latency, (M) resource management, (R) reliability.

**Table 2:** WiFi 7 low-latency use cases.

Sector and Use Case		Requirements	
	Latency (ms)	Reliability (%)	Throughput (Mbps)
**Multimedia**			
Real-time high-quality video streaming	3–10	>99.9	5–25
Virtual Reality	10–20	>99.9	25–500
Augmented Reality	1–50	>99.99	1–200
Real-time pro gaming	5–50	>99.9	>3
Cloud gaming	5–50	>99.9	10–35
**Health care**			
Telediagnosis, telemonitoring, and telerehabilitation	50–200	>99.9	0.5–5
Telesurgery	1–10	>99.9999	∼10
Exoskeletons and prosthetic hands	5–20	>99.999	0.2–1
**Industrial**			
Process automation	1–50	>99.99	0.1–5
Human machine interface	50–200	>99.9	∼1
Tactile/Haptic technology	1–5	>99.999	∼1
**Transport**			
Real-time traffic information	40–500	>99	0.1–1
Autonomous vehicle, automated guided vehicle, and drone control	10–100	>99.9999	1–5
Remote-controlled vehicle with video	10–100	>99.99	∼10

**Table 3:** Main simulation parameters.

**Deployment Parameters**	**B**	**Value**
*R*	Radius of the circular hall	15 m
$N_{\mathrm{AP}}$	Number of APs	1
$N_{\mathrm{BE}}$	Number of STAs requesting BE traffic	{15, 20, 25, 30}
$N_{\mathrm{TS}}$	Number of STAs requesting TS traffic	5
$p_{\mathrm{AP}}$	AP position	(0,0)
$p_{\mathrm{STA}}$	STA position	randomly selected
*d*	Distance between any STA and the AP	<15 m
*k*	Number of iterations per configuration	1000
**PHY & MAC Parameters**	**Description**	**Value**
$f_{c}$	Operating frequency	5 GHz
BW	Channel bandwidth	40 MHz
SS	Number of spatial streams	1
$P_{t}$	AP Transmission power	20 dBm
*S*	STA sensitivity	−90 dBm
$PL_{\mathrm{TMB}}\left(d\right)$	TMB path loss model	see [61]
$Na_{\mathrm{BE}}$	Number of BE aggregated packets	32
$Na_{\mathrm{TS}}$	Number of TS aggregated packets	16
$T_{\mathrm{ov}}$	Preemption overhead time	20 μs
$N_{f}$	Number of fragments of a preemptable packet	variable
**Traffic Parameters**	**Description**	**Value**
$L_{\mathrm{BE}}$	BE packet length	12,000 bits
$L_{\mathrm{TS}}$	TS packet length	4096 bits
–	Packet arrival process	Poisson
$B_{\mathrm{BE}}$	BE traffic per user	2 Mbps
$B_{\mathrm{TS}}$	TS traffic per user	5 Mbps
**Application Parameters**	**Description**	**Value**
$t_{\mathrm{TS}}$	Required TS packet latency	<5 m

## Data Availability

Not applicable.
